# General Anesthesia for Lumbar Puncture and Bone Marrow Aspiration /Biopsy in Children with Cancer

**Published:** 2013-04-22

**Authors:** A Ghasemi, M Gharavi Fard, AR Sabzevari

**Affiliations:** 1 Assistant Professor of Pediatric Hematology and oncology, Faculty of Medicine, Mashhad University of Medical Sciences, Mashhad, Iran.; 2 Associate Professor of Anesthesiology, Dr Sheikh Children Hospital, Faculty of Medicine, Mashhad University of Medical Sciences, Mashhad, Iran.; 3 Assistant Professor of Anesthesiology, Surgical Oncology Research Center, Dr Sheikh Children Hospital, Faculty of Medicine, Mashhad University of Medical Sciences, Mashhad, Iran.

**Keywords:** Anesthesia, General, Spinal Puncture, Biopsy

## Abstract

**Background:**

Multiple procedures (Lumbar puncture and bone marrow aspiration /biopsy) cause pain, stress, depression and etc for the patients and their families. Various methods have been recommended for pain reduction during invasive procedures. The aim of this study is to report the complications following general anesthesia.

**Materials and Methods:**

In this prospective observational study, two hundred and two children with cancer were enrolled. All patients received propofol 2.5 mg /kg and fentanyl 1 µg/kg. After adequate anesthesia, procedures were performed by a pediatric oncologist. All anesthesia complications were classified into two groups: Intraoperative and Postoperative complications. Complications which were recorded include: abnormal age-specific bradycardia (≤20 × baseline), decrease in arterial oxygen saturation (≤90%), laryngospasm, vomiting, agitation, headache, hypothermia (<35 C°), hyperthermia (>37/8 C°), signs of allergy, traumatic LP (bloody), and unusual local bleeding.

**Results:**

In this study, 118 males and 84 females underwent 623 general anesthetic procedures with a median of 3 procedures per patient. Intraoperative period complications occurred in 48 of total 623 procedures (7.7 %). The most common complications were traumatic LP, bradycardia and decrease in arterial oxygen saturation which occurred in 25, 6 and 6 cases, respectively. Postoperative period complications occurred in 74 (11.9%) cases. The most common complications were vomiting, agitation and headache, decrease O_2 _saturation and bradycardia.

**Conclusion:**

General anesthesia by propofol and fentanyl may be a good choice for short-term painful procedures in children undergoing treatment for bone marrow aspiration/biopsy and intratechal injection.

## Introduction

Multiple procedures (such as bone marrow aspiration and lumbar punctures) performed in children in pediatric oncology department. These procedures cause lots of pain for children and bring lots of stress for the patients and their families, particularly when these procedures are repeated many times during the diagnosis or treatment period (1). These procedures can also lead to depression and other psychiatric disorders (2). In a retrospective study of childhood cancer survivors, invasive procedures have been reported as the most difficult part of treatment, leading to posttraumatic stress symptoms in some long term survivors (3). 

As children do not habituate to pain, general anesthesia is recommended for all bone marrow biopsies. General anesthesia or combination of analgesia and sedative drugs during painful procedures in pediatric oncology is recommended by The World Health Organization and the American Academy of Pediatrics (AAP). (8. 9, 10)

Currently, various methods have been recommended for pain reduction during invasive procedures in this group of patients. These methods include: effective education for parents, preparation of the child for the procedure, cognitive behavioral therapy, sedation and general anesthesia (4). 

General anesthesia is defined as a drug-induced loss of consciousness when child is not arousal, even by painful stimulation. Children should be anesthetized by an anesthesiologist and in an operating room because some complications such as depressed ventilation, drug induced depression of neuromuscular function or cardiovascular dysfunction may occur and patient may need ventilation assistance (4). 

One of the common agents used for general anesthesia in children is propofol. This agent is administered intravenously and can induce rapid anesthesia when administered slowly over at least one minute. This agent causes a low incidence of nausea, vomiting and agitation during recovery. Another agent, fentanyl may be used in combination with propofol to alleviate pain, as propofol does not have analgesic properties. They also cause less adverse effects and shorter recovery time when used in combination (5, 6, and 7).

As mentioned above, general anesthesia is recommended for all invasive procedures in pediatric patients with cancer. In this study, we will report the complications following general anesthesia for lumbar puncture and bone marrow aspiration /biopsy in children with cancer.

## Materials and Methods

This is a prospective observational study which was held in pediatric oncology ward of Dr. Sheikh children hospital in Mashhad University of Medical sciences from Apr 2010 to Sep 2011. 

Two hundred and two children with cancer enrolled in our study (all of patients with inclusion criteria). Inclusion criteria was defined as all patients with cancer between 6 months to 14 years old ages who needed interventions such as Bone marrow aspiration (BMA), Bone marrow biopsy (BMB) and/or Intrathecal (IT) and patients with American Society of Anesthesiology physical status (ASA-ps) I-II. Excluding criteria were ASA-ps III-V, history of recent head injury; neurological abnormality, cardiopulmonary disease, drug allergies, and their parents were not willing to give informed consent. During diagnostic or therapeutic processes, whenever these patients needed lumbar puncture (LP) and/or BMA and BMB. Two hundred and two patients were enrolled in this study and 623 diagnostic or therapeutic procedures including LP and/or BMA, and BMB were performed totally. Prior to all procedures, all patients were interviewed by an expert nurse to get medical history of their disease and also any familial history. Complete blood count, PT, PTT and BT were measured performed. Information about patients' age, weight, ASA-ps score and disease diagnosis were recorded in their files. Anesthetic drugs and type of venous access were determined before the procedure.

All patients received propofol 2.5 mg /kg and fentanyl 1 µg/kg by a trained anesthesiologist. After adequate anesthesia was gained, procedures were performed by a pediatric oncologist.

All anesthesia complications were classified into two main groups: intraoperative complications are those occurred from the time of starting anesthesia to completion of surgical procedure and transferring the patients to the recovery room. Postoperative complications refer to those complications which occur while the patient is still in the recovery room. Complications which were recorded include: abnormal age-specific bradycardia (≤20 × baseline), decrease in arterial oxygen saturation (≤90%), laryngospasm, vomiting, agitation, headache, hypothermia (<35 C°), hyperthermia (>37/8 C°), signs of allergy, traumatic LP (bloody), and unusual local bleeding. All of these complications were recorded by an expert nurse who did not participate in the anesthesia process or LP/BMB/BMA procedures. All Patients remained in the operating room until they gained their consciousness. All patients had cardiopulmonary monitoring, pulse oximetry, blood pressure and temperature monitoring during all procedures.

This hospital is a referral center for pediatric surgery and anesthesiology in Khorasan province and these procedures were performed electively under general anesthesia at an outpatient unit in the operating room.

All anesthetic equipments and medications needed for life support were available. All procedures in patients were carried out under venous puncture. The patients had peripheral or central venous access and the anesthesia induction was performed with intravenous drugs. Anesthesia was inducted using propofol and fentanyl. 

All patients were discharged when they gained their consciousness completely and all vital signs went back to their normal values. 

Laryngospasm was transient and relived with positive pressure with mask and O2 support.

Decrease in arterial O_2_ saturation was mild (lowest O2 saturation was 89%) and relived with O2 support. 

Approval of the hospital ethics committee was obtained before the study and informed consent was signed by the parents of all patients. 

**Statistical Analysis**

The prevalence of these statistical factors was determined by SPSS v.15. Confidence interval in our study was 95% for all statistical tests.

## Results

Two hundred and two patients, consisting of 118 males and 84 females, underwent 623 general anesthetic procedures with a median of 3 procedures per patient. Diagnosis of our patients was hematologic and solid tumors. The mean age of patients was 5.5 ± 4.2 years.

Prior to the procedures, patient's cancer type had been diagnosed. One hundred and forty five patients had hematologic malignancies and 57 patients had solid tumors. The patient's characteristics are presented in table I.

Intraoperative complications: 

Intraoperative period complications occurred in 48 of total 623 procedures (7.7 %). The most common intraoperative period complications were traumatic LP, bradycardia and decrease in arterial oxygen saturation which occurred in 25, 6 and 6 cases, respectively (52.1%, 12.5%, and 12.5 %). 

Postoperative complications: 

Postoperative period complications occurred in 74 (11.9%) cases. The most common complications in the postoperative period were vomiting, agitation and headache which happened in 25, 21 and 11 cases, respectively (33.8%, 28.4%, and 14.9%).

**Table I:** Demographic variables and type of procedures in all of patients.

**sex**	**n**		**%**
**Male**	118		58.4
**female**	84		41.6
**procedures**			
**BMA**	200	32.1	
BMA/LP	131		21
**BMA/BMB**	12		2
**BMA/LP/IT**	6		1
**LP**	19		3
**LP/IT**	255		40.9
**cancer type**			
**hematologic**	145		72
**solid**	57		28

**Table II:** Details of procedure complications

**Complication**	**Intraoperative**		**Postoperative**	
	**N**	**%**	**N**	**%**
**Bradycardia**	6	12.5	0	0
**Laryngospasm**	4	8.3	0	0
**Decrease in arterial O**_2_ ** saturation**	6	12.5	2	2.7
**Vomiting**	0	0	25	33.8
**Agitation**	5	10.4	21	28.4
**Headache**	0	0	11	14.8
**Hyperthermia**	0	0	5	6.8
**Hypothermia**	0	0	3	4
**Allergy**	0	0	0	0
**Traumatic LP**	25	52.1	0	0
**Local bleeding**	2	4.2	7	9.5
**Total**	48	100	74	100

## Discussion

Various methods have been offered to reduce pain during invasive procedures in children undergoing cancer treatment. As these procedures bring pain and anxiety for children and their family. Implication of an effective pain reducing method seems necessary (11; 12). As we mentioned before, general anesthesia is currently recommended as the preferred method to reduce pain in patients undergoing lumbar puncture, bone marrow aspiration and bone marrow biopsy (8; 9).

In our study, we had 202 patients already diagnosed with cancer who underwent 623 procedures. We chose propofol in combination with fentanyl to induce anesthesia according to some literatures that have shown that this combination has a low rate of adverse effects and a shorter recovery time (5, 6,7). 

The most common complication during the intraoperative period was traumatic LP and in the postoperative period was vomiting followed by agitation.

This is in accordance with another study done by Meneses at al. The rate of complications in their study was similar to our study with traumatic LP being the most common complication during the intraoperative period and vomiting, agitation, headache being the most common complications in the postoperative period (13). Traumatic LP was associated with the LP procedure and not due to general anesthesia and it is possible 

that we could have higher rates if the patients were not sedated.

By considering all the procedures we found that complications in 20% of our cases were minor and 

did not have serious or life threatening complications. This rate is in accordance with the literature (14; 15). 

Although most of these complications can be considered as normal reactions, we recommend more investigation for a better understanding of the cause of these complications.

## Conclusion

We can conclude that general anesthesia by propofol and fentanyl is a good choice for short-term painful procedures in children undergoing treatment for malignancies.
